# Outcomes of BCG Induction in High-Risk Non-Muscle-Invasive Bladder Cancer Patients (NMIBC): A Retrospective Cohort Study

**DOI:** 10.7759/cureus.957

**Published:** 2017-01-05

**Authors:** Muhammad T Pirzada, Rashid Ghauri, Monis J Ahmed, Muhammad F Shah, Irfan ul Islam Nasir, Jasim Siddiqui, Irfan Ahmed, Khurram Mir

**Affiliations:** 1 Department of Surgical Oncology, Shaukat Khanum Memorial Cancer Hospital & Research Centre; 2 Department of Surgery, Mediclinic City Hospital, Dubai, UAE

**Keywords:** non-muscle-invasive bladder cancer (nmibc), bacillus calmette-guerin (bcg), recurrence, progressio

## Abstract

Non-muscle-invasive bladder cancer (NMIBC) is categorized into high-risk and low-risk groups. Although, bacillus Calmette-Guerin (BCG) is the recommended adjuvant therapy of high-risk bladder tumor, optimal schedule (induction versus maintenance) of this therapy is a subject of debate. The objective was to evaluate outcomes of induction BCG in high-risk NMIBC patients at Shaukat Khanum Memorial Cancer Hospital & Research Centre, Pakistan and retrospective cohort study conducted in the department of urology, Shaukat Khanum Memorial Cancer Hospital & Research Centre, Pakistan. Three-year disease-free survival and progression-free survival was the main outcome measure. Data of 68 high-risk (Ta and T1 with G3 or high-grade subtype) bladder cancer patients who underwent transurethral resection followed by six-weekly intravesical BCG instillation was included in the study. Recurrence was described as biopsy-proven bladder cancer; whereas the presence of muscle invasion was considered as progression. Disease-free survival and progression-free survival were defined as time intervals elapsed between the starting date of BCG instillation and recurrence or progression, respectively. Kaplan-Meier curve was employed to estimate the three-year study end-points. Disease-free survival at three years was observed to be 66.2% and progression-free survival at 86.8%. The use of induction BCG alone for high-risk patients of NMIBC is a viable option both in terms of effective disease-free and progression-free survival rates.

## Introduction

Transitional cell carcinoma (TCC), the commonest type of bladder cancer, has a heterogeneous clinical spectrum, disease progression, therapeutic modalities and prognosis [1]. Approximately, 75% of the cases manifest as superficial lesions involving the mucosa and submucosa [2]; however, diverse morphological and histopathological features of these lesions have been defined [3]. On the basis of histopathology, superficial lesions are stratified from papillary urothelial neoplasms of low malignant potential (PUNLMP) to high-grade urothelial carcinomas, whereas morphologically, they are separated into three subtypes: papillary tumors confined to mucosa (Ta), papillary or nodular variety with invasion into the lamina propria (T1), and ‘flat tumors’ confined to urothelium (Tis or carcinoma in situ (CIS) [3]. As these tumors are amenable to transurethral resection, therefore, they are categorized as non-muscle-invasive bladder cancer (NMIBC) [4].

Although, transurethral resection is a well-recognized initial treatment modality for all NMIBC, institution of subsequent adjuvant intravesical therapy to prevent tumor recurrence and progression to muscle invasive category depends on risk groups derived from numerous prognostic factors [5]. It has been documented that tumor progression and recurrence rates after transurethral resection are substantial in high-risk group as compared to low-risk group [6]; even so, these untoward outcomes have been reduced by intravesical chemo- and/or immunotherapy [7-8]. While the appropriate choice amongst risk groups had been a subject of debate in 2006, European Association of Urology standardized the adjuvant intravesical therapy for the management of superficial bladder cancers according to risk groups stratification: transurethral resection followed by instillation of intravesical chemotherapy (mitomycin-C) in cases of low-risk groups and use of BCG (bacillus Calmette-Guerin) in cases of high risk groups [9].

The archetypal schedule, known as induction therapy, in high-risk group bladder cancer patients includes six-weekly intravesical instillation of BCG [4]. In order to enhance recurrence-free and to some extent progression-free survival, a number of studies suggest the addition of maintenance therapy of BCG. Malmstrom and colleagues showed a 28% cumulative increase in the risk of recurrence in trials where BCG maintenance was not used [10]. Lamm and associates found statistically significant difference in median recurrence-free survival in cohort of patients who received BCG maintenance therapy as compared to no maintenance group [11]. On the similar note, Bohle and Bock demonstrated a prevention of tumor progression with the provision of BCG maintenance therapy [12].

Several studies also demonstrate the efficacy of BCG induction therapy alone both to delay tumor progression and to improve the five-year disease-free survival [13,15]. A study of 1021 patients who underwent BCG induction therapy alone, evaluated five-year recurrence-free and tumor-free survival rates and revealed 46% recurrence-free and 89% progression-free survival rates associated with classical BCG regimen [13]. Likewise, Koga and colleagues showed superior efficacy of BCG induction in their randomized controlled trial [14].

Hitherto, a large body of controversial reports and contradictory discursive evidence has been observed in the literature regarding the optimal regimen of adjuvant BCG therapy in high-risk bladder cancer. Although, published guidelines recommend maintenance BCG for one to three years [9,15], substantial non-adherence to this recommendation as a consequence of BCG cumulative toxicities has been evident [16-17]. Therefore, keeping the view of poor patient compliance on account of side effects, empirical BCG induction is the principal adjuvant therapy in high-risk bladder cancers at Shaukat Khanum Memorial Cancer Hospital and Research Center, Pakistan.

The purpose of this study was to evaluate the outcomes of induction BCG in high-risk NMIBC patients at Shaukat Khanum Memorial Cancer Hospital & Research Centre, Pakistan. Both progression-free and disease-free survivals were estimated prospectively by analyzing retrospective data. The data will provide supporting evidence in scientific literature about the ongoing debate of optimal BCG schedule but also valuable in the revision of protocol for high-risk NMIBC patient’s management at local level. Informed consent was obtained from the patient for this study

## Materials and methods

This retrospective review of the database of patients having bladder cancer at Shaukat Khanum Memorial Cancer Hospital & Research Centre, Pakistan, was accomplished from January 2008 to Dec 2012 after approval of Institutional Review Board (IRB).

### Patient selection

Clinical information of all patients of both sexes who underwent transurethral resection followed by intravesical BCG induction therapy between January 2008 to December 2012 having following parameters were included in the study: Ta and T1 tumor categories, high-grade, between 50 to 80 years of age. Patients who underwent transurethral resection followed by intravesical instillation of chemotherapy or combination of both chemotherapy and BCG, previously treated with BCG therapy, had recurrent tumors, isolated carcinoma in situ (CIS), associated upper urinary tract urothelial tumors, and non-urothelial bladder cancer and patients with inconclusive histopathology due to absence of muscle layers in specimen were excluded.

### Procedure details

The included patients had undergone transurethral resection of bladder tumor by a consultant urologist. After two weeks, TICE BCG in a dose of 500 mIU diluted in 50 ml 0.9% saline was given intravesically once a week for six weeks. The instilled amount was retained in bladder for an hour. Tumor recurrence and progression were evaluated on check cystoscopy scheduled at every three months for three years after primary resection of bladder cancer.

### Data collection

All clinical records were recorded into proforma designed for the study. Data collection process included: socio-demographic status, date of first cystoscopy and transurethral resection, tumor category, tumor extent, tumor size, associated CIS, BCG therapy compliance, and surveillance status. The tumor surveillance incorporated regular check cystoscopies after primary resection and BCG induction. The occurrence of biopsy-proven bladder cancer was considered as recurrence, whereas presence of muscle invasion on histopathology was described as progression. Disease-free survival and progression-free survival were defined as time intervals elapsed between the starting date of BCG instillation and recurrence and progression, respectively. Outcome end point was three-year disease-free and progression-free survival. Data which showed no events throughout the surveillance period, lost to follow-up patients and those who discontinued BCG were considered as censored events.

### Data analysis

The Statistical Package for the Social Sciences (SPSS) version 19 was utilized for all statistical analysis. Mean SD was employed for quantitative variable. Frequencies and percentages were utilized to summarize qualitative variables. Times to events (i.e. recurrence and progression) were calculated considering the start date of BCG as time zero. Kaplan-Meier curve was employed to estimate the disease-free survival and progression-free survival. Multivariate cox proportional hazard model was used to adjust for the potential confounding effect of explanatory variables on end-point variables with the p-value < 0.05 taken as statistical significance. The results were also described in hazard ratio (HR) and 95% confidence interval (CI).

**Table 1:** Patient characteristics CIS= carcinoma in situ

Characteristics	n=68	%
Age		
< 60 years	28	41.2
> 60 years	40	58.8
Mean (SD) age = 62.66 (12.05)		
Sex		
Male	60	88.2
Female	08	11.8
Tumor category		
Ta	03	4.4
T1	65	95.6
Tumor extent		
Single	40	58.8
Multiple	28	41.2
Tumor size, No		
< 3 cm	40	58.8
> 3 cm	28	41.2
Associated CIS		
Yes	03	4.4
No	65	95.6

## Results

**Figure 1:**
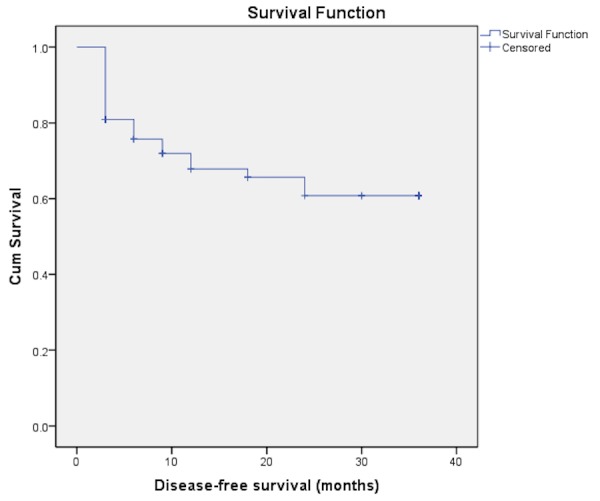
Disease-free survival

**Figure 2:**
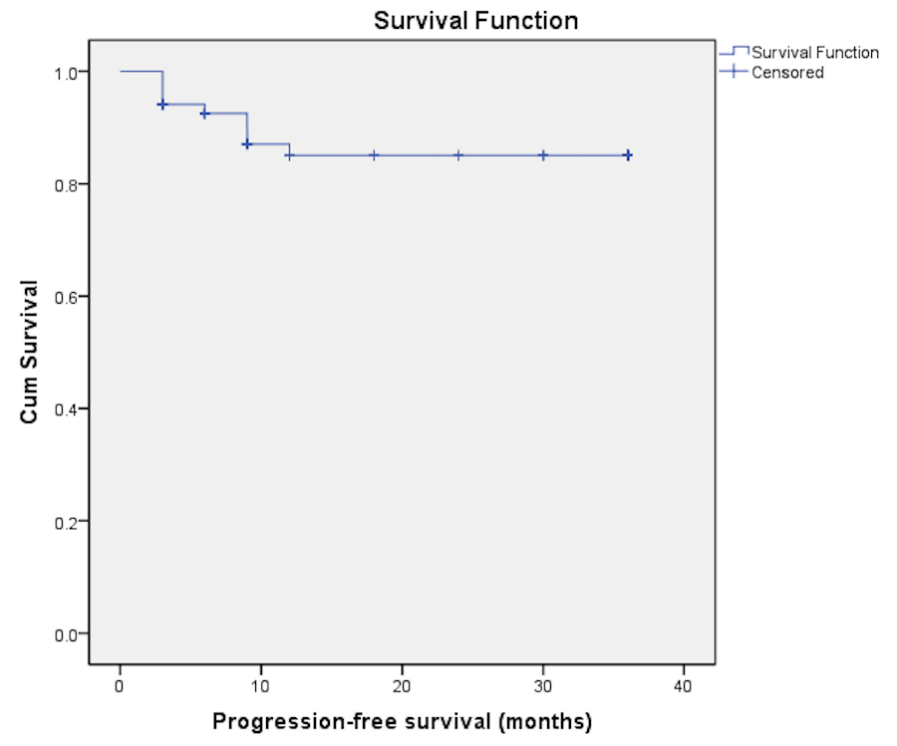
Progression-free survival

A total of 68 patients fulfilling the inclusion criteria were identified by retrospective analysis of the registered cases. The mean age (SD) of the study group was 62.66 + 12.05 years. 41.2% were < 60 years of age, and 88.2% were male. Three patients had T_a_ disease and 65 (95.6%) had T_1_ disease. Forty (58.8%) patients had a single lesion and the same number had a tumor size less than 3 cm.  Associated CIS was found in three (4.4%) of the patients (Table 1). With regards to disease recurrence 24 patients completed follow-up while the rest were censored. Three-year disease-free survival rate was found to be 66.2% in our patients (Figure 1). The progression-free survival estimated at our center was 86.8% (Figure 2). Tumor recurrence was noted in 10 (25%). Median follow-up was 24.7 months. Multivariate analysis of factors affecting disease-free survival showed that tumor extent was the only factor significantly influencing disease-free survival independently (Table 2). None of the variables independently affected progress free survival.

**Table 2:** Multivariate analysis of variables for disease-free and progression-free survival HR = Hazard ratio; CI = Confidence interval, CIS= carcinoma in situ

Variables	HR (95% CI)	p-value
Disease-free survival		
Age (< 60 vs. > 60 yrs)	2.24 (0.97 – 5.18)	0.059
Sex (male vs. female)	1.03 (0.23 – 4.58)	0.964
Tumor category (Ta vs. T1)	0.52 (0.06 – 4.12)	0.531
Tumor extent (single vs. multiple)	0.42 (0.18 – 0.98)	0.046
Tumor size (< 3 vs. > 3cm)	0.76 (0.32 – 1.80)	0.532
Associated CIS (yes vs. no)	0.48 (0.06 – 3.76)	0.482
Progression free survival		
Age (< 60 vs. > 60 yrs)	1.39 (0.66 – 5.33)	0.624
Sex (male vs. female)	0.95 (0.21 – 4.28)	0.951
Tumor category (Ta vs. T1)	0.49 (0.06 – 3.97)	0.507
Tumor extent (single vs. multiple)	1.55 (0.30 – 6.13)	0.532
Tumor size (< 3 vs. > 3 cm)	0.67 (0.16 – 2.83)	0.580
Associated CIS (yes vs. no)	0.58 (0.08 – 4.55)	0.608

## Discussion

Bladder cell carcinomas are the fourth most common cancer affecting the American males and accounts for 60,000 new cases in the United States alone [18-19]. Approximately 80% of these cancers are non-muscle invasive bladder cancer (NMIBC). The reported burden in Pakistan is 5.6% of all registered cancers [20].

A varied range of treatment options has been proposed, tested and rejected over the course of many years for optimal management of this clinical entity. Transurethral resection, combination of resection with chemotherapy and intravesical instillation of immunotherapeutic agents have all been proposed to varying degrees of success and the debate still rages on.

Bacillus Calmette-Guerin (BCG) was initially developed to treat tuberculosis. Pearl in 1929 was the first to report its anti-neoplastic activity [19]. Zbar, et al. were the first to report its use as an intra-dermal agent for treatment of cancers [21]. Since then the use of BCG has been established as a treatment for bladder cancers though the optimal methodology is still controversial.

The current debate focuses on whether induction therapy of BCG alone is sufficient or should the maintenance therapy be pursued. One of the main limitations associated with the use of this agent is the local and systemic side effects which range from cystitis, epididymitis, prostatitis to lung infection, liver toxicity and sepsis, these side effects are dose dependent [22]. Hence a fine balance between the achievement of optimal clinical outcome and limiting the toxic side effects is the target that will be achieved by using a dose that is just low enough to cause minimum side effects yet not be compromising the clinical outcomes.

We at our institute, therefore decided to follow induction therapy as the methodology for treatment of high risk NMIBC. Using this regimen protocol we found the three-year disease-free survival in our patients was 66.2% and the progression-free survival for the same period was 85%. Herr, et al. in their study of 816 patients who got induction chemotherapy showed a two year and five year disease-free survival of 73% and 46% respectively. Their calculated progression-free survival was 89% [13]. Despite the fact that our study sample is smaller but the results that we have achieved are comparable to the observations of Herr and colleagues.

A recent study was published evaluating maintenance BCG therapy [23]. The authors observed a three-year recurrence free survival of 75.3% and disease-free survival of 96.1%. However, they reported a high complication rate of 81.5%. Though this study demonstrated better disease free and recurrence free outcomes, the results need to be analyzed carefully as the sample size is quite low in 27 patients. Also the high complication rates re-demonstrate the limitation of maintenance therapy. Another recent meta-analysis with a pool of 1120 patients who received full dose maintenance therapy showed a recurrence rate of 33.3% [22].This is slightly lower but still comparable with the outcome achieved at our institution. 

A recently published multi-centre study that randomly allocated patients to no maintenance versus maintenance arm showed 33.5% and 38.5% recurrence rates. This study had 195 patients in the former arm and 202 in the latter. They concluded that maintenance therapy did not lead to a decrease in recurrence or progression. They reported that 20 patients in the maintenance arm had to stop treatment because of side effects and only five faced this situation in the no maintenance arm [24].

The following factors should be considered while interpreting our results: this study is based on retrospective review of database. Although, attention was given to methodological rigor by incorporating explanatory variables and analyzing confounding effect of these on endpoint measures, various biases related to operative and BCG instillation techniques cannot be controlled. Secondly, the study has a relatively short follow-up time. Therefore, both the disease-free and progression-free survival rates may not be as precise as other longer surveillance published series [13-14].

## Conclusions

The use of induction BCG alone for high-risk patients of NMIBC is a viable option. The disease-free and progression-free survival rates at our institution using this option is comparable with the internationally published data.
